# The impact of sampling bias on preferences for skewed distributions in decisions from experience

**DOI:** 10.1073/pnas.2418336122

**Published:** 2025-04-07

**Authors:** Yonatan Vanunu, Ben R. Newell

**Affiliations:** ^a^Coller School of Management, Tel Aviv University, Tel Aviv 6997801, Israel; ^b^Sagol School of Neuroscience, Tel Aviv University, Tel Aviv 6997801, Israel; ^c^School of Psychology, UNSW Sydney, Sydney, NSW 2052, Australia

A recent PNAS article proposes a tallying mechanism to reconcile the discrepancy between a preference for right-skewed reward distributions in economic theories and left-skewed distributions in empirical studies on decisions-from-experience (1). The authors suggest that choice is determined by how often one option displays a larger reward than the other—*the frequent winner effect*. However, they claim that there is a choice bias toward right-skewed distributions when neither option is the frequent winner.

These findings highlight a novel tallying mechanism that diverges from traditional learning models in decisions-from-experience. However, a choice bias for right-skewed distributions contradicts recent findings showing a robust preference for left-skewed distributions, even when the “frequent winner” is controlled for (2). In that study, participants were presented with an array of rewards and tasked with choosing between taking a gamble with equal probability of winning one of the rewards or a known certain payoff, with a balanced number of rewards above and below the certain payoff. The preference for left-skewed distributions was explained by an incomplete sampling process based on a subset of the largest rewards in the display. Notably, the two studies differ in demands on cognitive capacity: The former (1) displays 60 rewards per trial, while the latter (2) presents 8.

A choice mechanism based on a subset of outcomes is common in decisions-from-experience. Research has shown that the experience–description gap (3, 4) can be explained by a process relying on small memory samples, where rare outcomes are often underrepresented (5–8), but extreme outcomes are likely included due to their memorability (9–10). Therefore, a preference for right-skewed distributions may stem from the subset of rewards recalled from memory, influenced by sequence length.

**Fig. 1.**
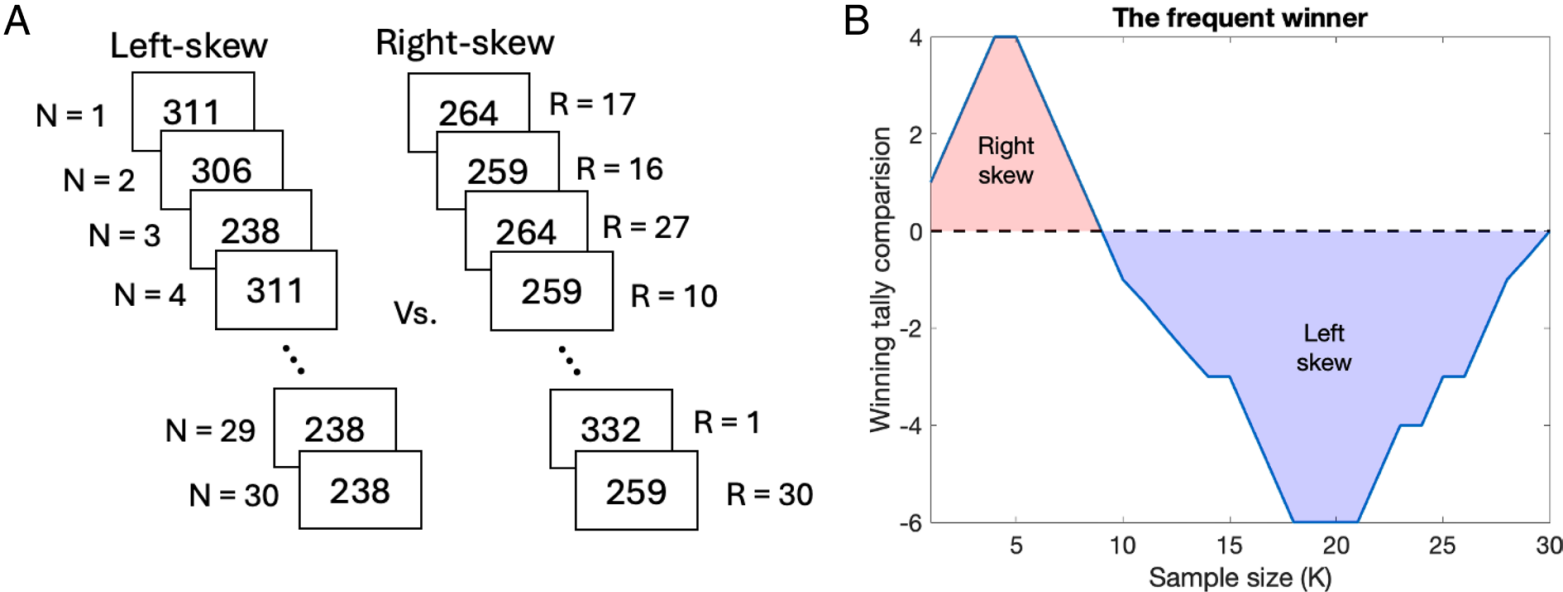
(*A*) Example display from the “broker game” in Study 3 (ref. 1), where participants viewed a sequence of 30 pairs of dividends from two stocks—one with a left-skew distribution and the other with a right-skew distribution—and were asked to choose from which stock they wished to obtain a dividend. Importantly, each stock had higher values in exactly half of the observations, ensuring the frequent winner across the sequence was balanced. R represents the value difference rank, ranging from 1 (the largest difference) to 30 (the smallest difference). Identical differences were ranked consecutively. N represents the temporal position in the sequence. (*B*) A simulation of the frequent winner comparison as a function of sample size *K*. If *K* = 1, the subsample contains the winner from the largest rank (R = 1), and as *K* increases, winners from the following ranks are included in the subsample until reaching the full sample at *K* = 30. A positive winning tally value in the figure (red area) indicates a preference for right-skew distributions over left-skew distributions, while a negative winning tally value (blue area) indicates the opposite.

To illustrate this notion, we simulated variations in the frequent winner as a function of sample size *K* when the frequent winner across the sequence was balanced (study 3 in ref. 1). We ranked the rewards in the sequences by the absolute difference between them (Fig. 1*A*), assuming that larger differences are more memorable. We then computed the frequent winner considering a subsample of the largest value differences, where *K* = 1 included the winner of the largest value difference, and as *K* increased, winners from the following ranks were included until reaching the full sample at *K* = 30. Findings show the right-skew distribution was the frequent winner if fewer than 10 winners from the largest ranks were included, while the left-skew distribution was the frequent winner if more than 10 were included (Fig. 1*B*).

We conclude that when capacity is challenged by sequence length, a preference for right-skewed distributions may emerge due to reliance on small samples containing the most memorable outcomes. However, with fewer outcomes to remember, more comprehensive sampling may favor left-skewed distributions. Thus, a preference for right-skewed distributions might be driven by a sampling bias. Future experiments could determine the specific impact of sequence length on skewness preferences, while controlling for other procedural differences between (1) and (2) (e.g., presence of a safe option).
